# Genetic diversity of *Plasmodium falciparum* isolates from uncomplicated malaria cases in Ghana over a decade

**DOI:** 10.1186/s13071-016-1692-1

**Published:** 2016-07-26

**Authors:** Nancy O. Duah, Sena A. Matrevi, Neils B. Quashie, Benjamin Abuaku, Kwadwo A. Koram

**Affiliations:** 1 Epidemiology Department, Noguchi Memorial Institute for Medical Research, College of Health Sciences, University of Ghana, P. O. Box LG581, Legon, Ghana; 2 Centre for Tropical Clinical Pharmacology and Therapeutics, School of Medicine and Dentistry, College of Health Sciences, University of Ghana, P. O. Box GP 4260, Accra, Ghana

**Keywords:** Genetic diversity, *Plasmodium falciparum*, *msp2*, Ghana

## Abstract

**Background:**

Genotyping malaria parasites to assess their diversity in different geographic settings have become necessary for the selection of antigenic epitopes for vaccine development and for antimalarial drug efficacy or resistance investigations. This study describes the genetic diversity of *Plasmodium falciparum* isolates from uncomplicated malaria cases over a ten year period (2003–2013) in Ghana using the polymorphic antigenic marker, merozoite surface protein 2 (*msp2*).

**Methods:**

Archived filter paper blood blots from children aged nine years and below with uncomplicated malaria collected from nine sites in Ghana were typed for the presence of the markers. A total of 880 samples were genotyped for *msp2* for the two major allelic families, FC27 and 3D7, using nested polymerase chain reaction (PCR). The allele frequencies and the multiplicity of infection were determined for the nine sites for five time points over a period of ten years, 2003–2004, 2005–2006, 2007–2008, 2010 and 2012–2013 malaria transmission seasons.

**Results:**

The number of different alleles detected for the *msp2* gene by resolving PCR products on agarose gels was 14. Both of the major allelic families, 3D7 and FC27 were common in all population samples. The highest multiplicity of infection (MOI) was observed in isolates from Begoro (forest zone, rural site): 3.31 for the time point 2007–2008. A significant variation was observed among the sites in the MOIs detected per infection (Fisher's exact test, *P* < 0.001) for the 2007 isolates and also at each of the three sites with data for three different years, Hohoe, *P* = 0.03; Navrongo, *P* < 0.001; Cape Coast, *P* < 0.001. Overall, there was no significant difference between the MOIs of the three ecological zones over the years (*P* = 0.37) and between the time points when data from all sites were pooled (*P* = 0.40).

**Conclusions:**

The diversity and variation between isolates detected using the *msp2* gene in Ghanaian isolates were observed to be profound; however, there was homogeneity throughout the three ecological zones studied. This is indicative of gene flow between the parasite populations across the country probably due to human population movements (HPM).

**Electronic supplementary material:**

The online version of this article (doi:10.1186/s13071-016-1692-1) contains supplementary material, which is available to authorized users.

## Background

Malaria parasite genetic diversity creates a great hindrance to vaccine development efforts and enhances antimalarial drug resistance. Genetic diversity occurs as a result of genetic recombinations from numerous allelic polymorphisms exhibited at various genetic loci and also through diversifying selection by immunity [1, 2]. It has been shown to be comparatively high in hyper-endemic areas than in low endemic areas [1, 2]. The level of antigenic diversity resulting in the multiplicity of infections varies from one malaria endemic region to another and even between countries. Such that the variant forms of the parasite exist at different frequencies in different geographic areas presenting different complexities of infection [3]. Parasite genetic diversity has been implicated in evolutionary fitness and consequently populations with high diversity have the ability to survive against ongoing interventions in malaria endemic areas thereby frustrating control efforts [4, 5].

The merozoite surface proteins 2 (*msp2)* is a polymorphic antigenic marker that has been used extensively to describe the diversity of parasite populations in many malaria endemic countries. *Msp2 *gene has two major allelic families, FC27 and ICI/3D7 based on the variable non-repeat sequences as well as the varying sizes of the tandem repeats in the central region [6, 7]. This parasite surface antigen plays a role in parasite invasion of the erythrocytes and due to the high polymorphism they exhibit, the parasite gains the ability to evade immune responses [8, 9]. Of the three marker genes, *msp1, msp2* and glutamine-rich protein (*glurp*), which have been endorsed by the WHO for use in distinguishing between recrudescence and new infection in recurrent infections during antimalarial drug efficacy investigations, the *msp2* marker is the most polymorphic and therefore the highest discriminatory and informative marker [9–13].

Ghana is a malaria endemic country with three different ecological zones with either perennial or seasonal transmission of malaria. The northern part of the country has Guinea savannah ecology, middle belt has forest ecology and the southern part has coastal savannah ecology. Seasonal transmission is observed in the northern part whilst the forest and the coastal savannah experiences perennial transmission. The vectors of transmission vary per the ecological zones such that *Anopheles gambiae* (*sensu stricto*) transmits the parasite in all 3 ecological zones, *A. melas* transmit in the coastal savannah zone, *A. arabiensis* and *A. funestus* (*s.s*.) transmits mostly in the Guinea savannah zone of the country. Transmission intensities are high with peaks observed during the wet season. Malaria accounted for 44 % of all outpatient clinic visits in 2013 and 22.3 % of all under-five deaths in Ghana [14]. The main control strategy is active case detection and treatment using artemisinin-based combination therapy (ACT). Other interventions include intermittent preventive treatment among pregnant women (IPTp), seasonal malaria chemoprevention (SMC), long lasting insecticide-treated nets (LLINs), and indoor residual spraying (IRS) [15].

Information on the diversity of malaria parasites in Ghana is scanty and in the search for an effective vaccine for the African malaria endemic region it is crucial to describe genetically, the parasite population structure over the years. This study therefore determined the genetic diversity of parasites in the country by detecting the presence of *msp2* alleles in *P. falciparum* isolates from uncomplicated malaria cases collected over ten years (2003–2013) from nine sentinel sites for monitoring antimalarial drug efficacy/resistance in Ghana. Findings from this study will serve as baseline data for future studies on parasite population structure and antimalarial drug resistance surveillance in the country.

## Methods

### Study sites

**Fig. 1:**
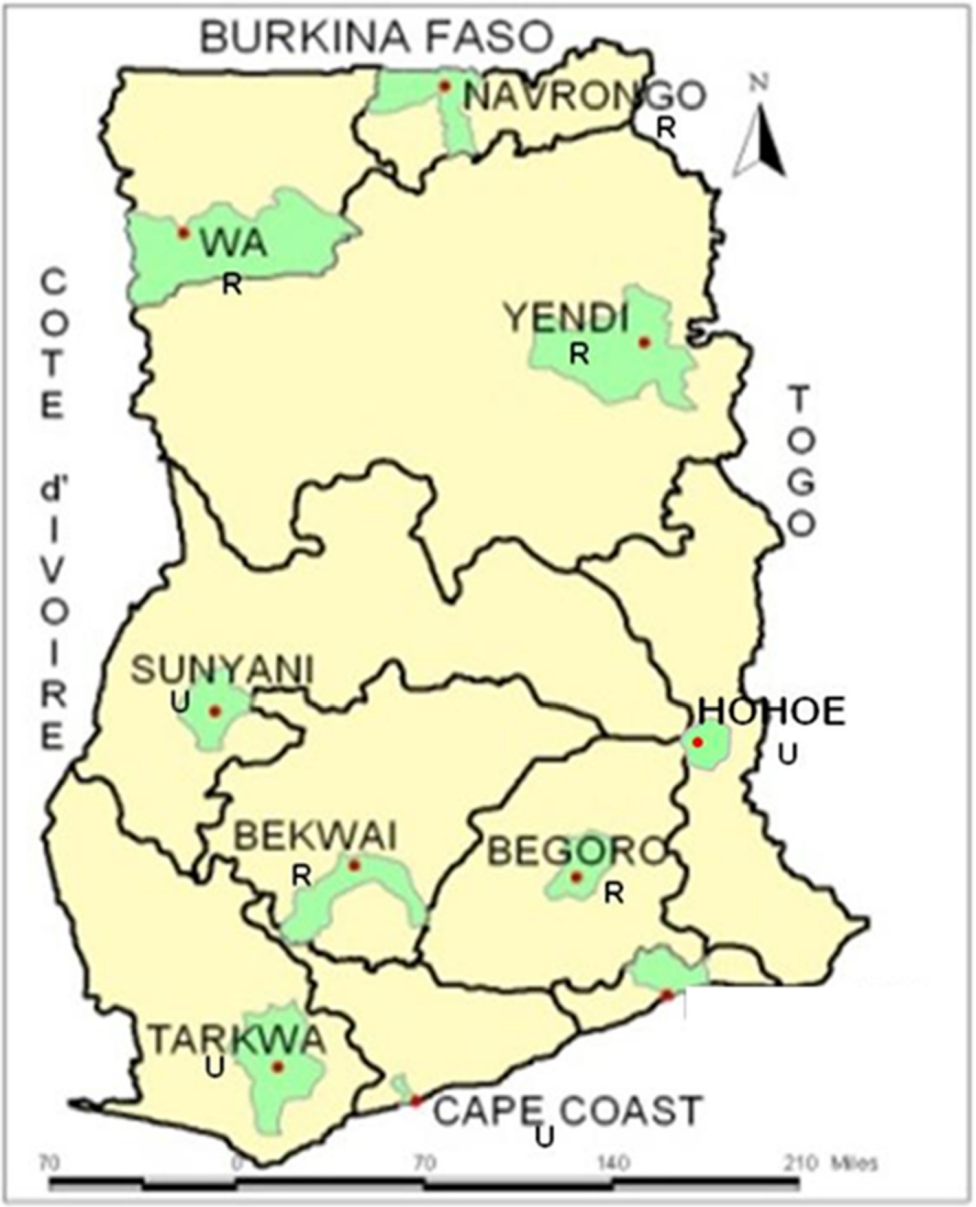
A map of Ghana showing the ten sentinel sites for monitoring antimalarial drug efficacy/resistance in the country. These sites are located in the three ecological zones of Ghana and were set up by a joint collaboration between Noguchi Memorail Institute fo Medical Research (NMIMR) and the National Malaria Control Programme (NMCP). *Abbreviations*: *R*, rural setting; *U*, urban setting

The archived samples used for this study were collected in 2003–2013 from nine out of the ten sentinel sites set up by the Noguchi Memorial Institute for Medical Research (NMIMR) and the National Malaria Control Programme (NMCP) for monitoring antimalarial drug resistance in the country. The description of these sites has already been published [16–22]. These sites were categorised into three ecological zones and urbanicity: Navrongo (rural; 10.9840°N, 1.0921°W), Wa (rural; 10.0601°N, 2.5099°W) and Yendi (rural; 9.4450°N, 0.0093°W) in the guinea savanna with seasonal malaria transmission; Begoro (rural; 6.3916°N, 0.3795°W), Bekwai (rural; 6.4532°N, 1.5838°W), Hohoe (urban; 7.1519°N, 0.4738°E), Sunyani (urban; 7.3349°N, 2.3123°W) and Tarkwa (urban; 5.3018°N, 1.9930°W) in the tropical forest with perennial malaria transmission; Cape-Coast (urban; 5.1315°N, 1.2795°W) in the coastal savanna with perennial malaria transmission. A map of Ghana indicating the sites is shown in Fig. 1.

### Study samples

Archived filter paper blood blots collected from children aged nine years and below with uncomplicated malaria from antimalarial drug resistance surveillance studies conducted in 2003 to 2013 and stored at room temperature were used [16–22]. These samples were collected after the parents or guardians of these children gave informed consent for their participation in the studies. Ethical approval for the study was given by the NMIMR IRB.

### Detection of *Plasmodium* species and *msp2* alleles by PCR

Parasite DNA from 880 filter paper blood blot samples was extracted using Qiagen DNA Blood Minikit (Qiagen, California USA). Parasite species detection using nested PCR was done following published protocols with minimal changes [23, 24]. Nested PCR was used for the detection of *msp2* alleles following recommended standardised protocols from the Worldwide Antimicrobial Resistance Network (WWARN) and World Health Organization (WHO) for the identification of parasite populations [10–12]. The primers for both primary and nested PCRs for the detection of the alleles of the *msp2* gene are from previously published protocols [10–12] (Additional file 1: Table S1).

### Data analysis

Sensitivity of PCR was determined from the number of PCR positives over the total number of samples analysed. Frequencies of alleles of the genes were determined for each sample by individual counts of PCR positivity. Multiplicity of infection (MOI) defined as the mean number of genotypes per infection was determined as the quotient of the total number of alleles per locus over the total number of PCR positive samples per locus. Differences in the MOI between time points and the ecological zones were determined by analysis of variance (ANOVA) whilst a difference in MOIs between the sites per time point was determined using Chi-square test and Fisher’s exact test.

## Results

A total of 880 samples from nine sites were typed for *msp2* alleles, FC27 and 3D7 for five different time points. Of the 880 samples analysed, 52 (6 %) were from 2003 to 2004, 209 (24 %) from 2005 to 2006, 372 (42 %) from 2007 to 2008, 89 (10 %) from 2010 to 158 (18 %) from 2012 to 2013. The contribution from each site to the total number of samples is shown in Table 1. The sensitivity of the PCR method for the typing of the alleles of *msp2* ranged from 46 to 100 %. It was observed that sensitivity was comparatively higher in the most recently collected samples 2007–2013. Therefore, data analysis was conducted with 711 PCR positive samples, 37 from 2003 to 2004, 136 from 2005 to 2006, 332 from 2007 to 2008, 73 from 2010 to 133 from 2012 to 2013 (Additional file 2: Table S2).

**Table 1:** The number of samples from each site and the time point

Sites	No. of samples (overall %)	Time points
Begoro	90 (10)	3 (2005–2006, 2007–2008, 2010)
Bekwai	82 (9)	3 (2005–2006, 2007–2008, 2010)
Cape-Coast	156 (18)	4 (2005–2006, 2007–2008, 2010, 2012–2013)
Hohoe	95 (11)	3 (2003–2004,2007–2008, 2012–2013)
Navrongo	187 (21)	5 (2003–2004,2005–2006, 2007–2008, 2010, 2012–2013)
Sunyani	79 (9)	2 (2005–2006, 2007–2008)
Tarkwa	18 (2)	1 (2007–2008)
Wa	84 (10)	3 (2005–2006, 2007–2008, 2010)
Yendi	89 (10)	3 (2005–2006, 2007–2008, 2010)

### Allele frequencies for *msp2*

Fourteen different alleles were detected for *msp2* gene by analysis of PCR products on agarose gels. The most frequent allele sizes which persisted in all the three ecological zones within the periods were FC27-400 and 3D7-600. All the genotypes in each isolate are shown in full in Additional file 3: Tables S3-S7). There was no significant difference between the allele frequencies of FC27 and 3D7 over the years and the trend analysis of allele frequencies was also not significant (*χ*^2^ = 2.484, *df* = 1, *P* = 0.115).

### Multiplicity of infection (MOI)

**Fig. 2:**
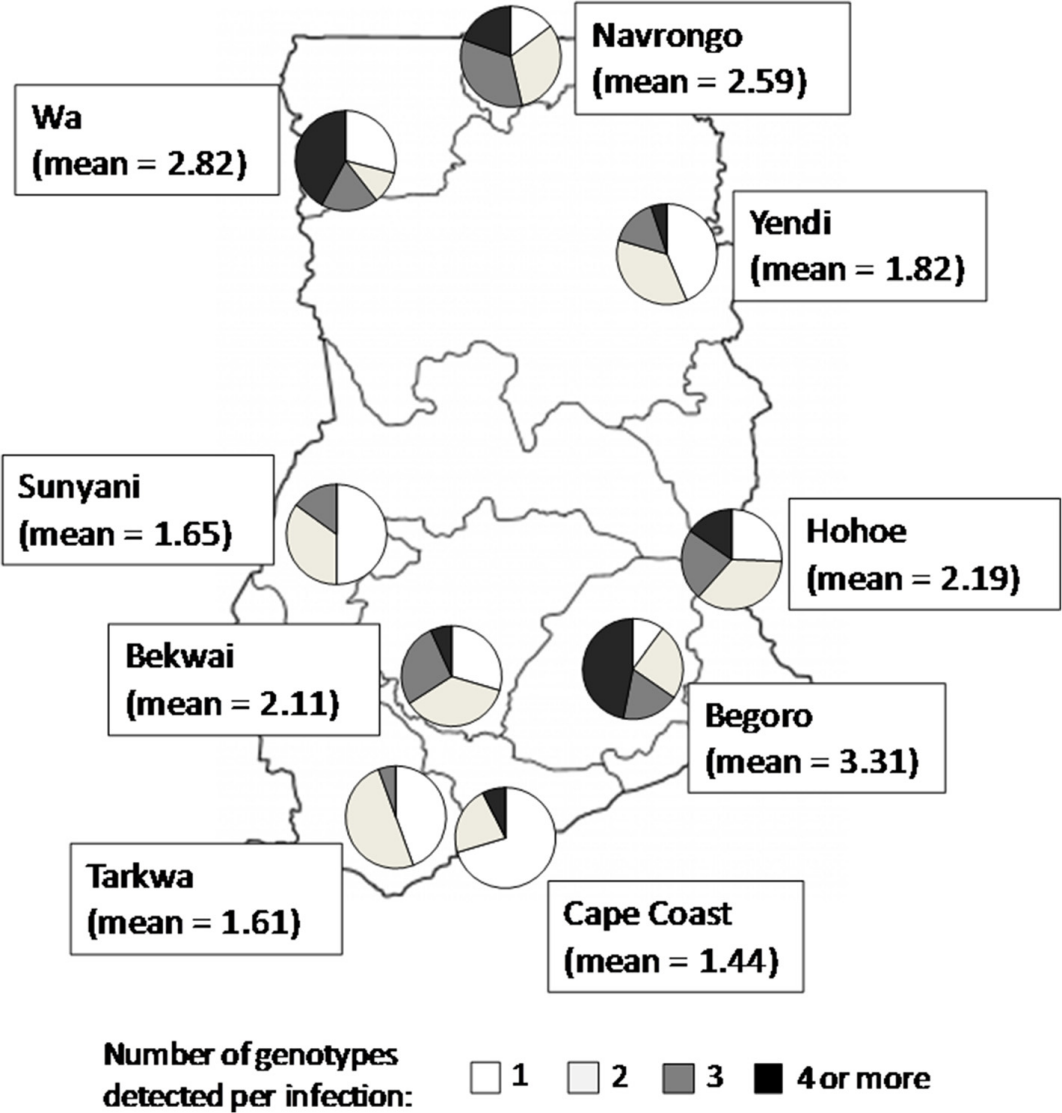
The distribution of *msp2* mean MOIs across the nine sites from isolates collected in 2007. Pie charts show the number of genotypes per infection, ranging from 1 to 4 genotypes per infection

**Fig. 3:**
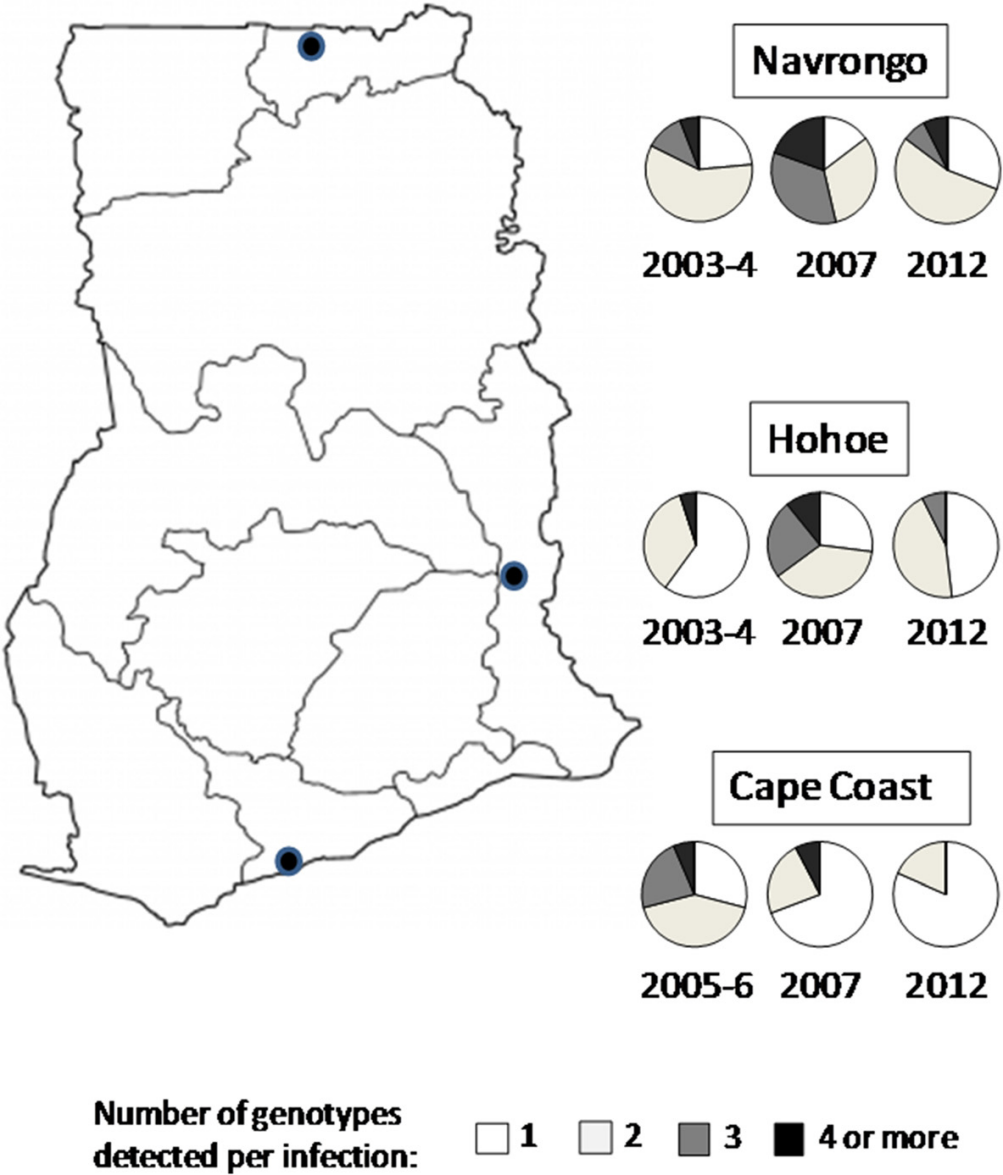
The *msp2* mean MOIs for three sites located in three ecological zones over three time points. There were significant differences in the MOIs between the years for each site. The pie charts depict the number of genotypes per infection ranging from 1 to 4 genotypes

The MOI defined as the mean number of genotypes per infection (Table 2) for each locus per time point was determined for every site, and geometric mean MOIs were computed for the different ecological zones and for the country (Table 2). The highest MOI was 3.31 in Begoro (forest zone) in 2007–2008 (Table 2). The geometric mean MOIs for all the ecological zones per time point are shown in Table 3. The MOIs ranged between 1.07–2.82, 1.40–3.31 and 1.13–2.03, respectively for Guinea savannah, forest and coastal savannah zones for all the time points. No significant difference was observed between the geometric mean MOIs of the ecological zones (Fisher’s exact test *P* = 0.370) and between the five time points when the data was pooled (Fisher’s exact test *P* = 0.405). Except for the isolates from 2007 which showed a significant variation among the sites in the distributions of numbers of alleles of *msp2* detected per infection (Fisher’s exact test, *P* < 0.001) (Fig. 2). There was also significant variation in the MOI across the years for three sites with data for three years using Fisher’s exact test; Hohoe, *P* = 0.03; Navrongo, *P* < 0.001; Cape Coast, *P* < 0.001 (Fig. 3).

**Table 2:** Multiplicity of infection (mean MOI) for all the time points at the sites

	Mean MOIs at time points				
Sites	2003–2004	2005–2006	2007–2008	2010	2012–2013
Begoro	–	1.65	3.31	1.64	–
Bekwai	–	1.72	2.11	1.40	–
Cape-Coast	–	2.03	1.44	1.31	1.13
Hohoe	1.50	–	2.18	–	1.59
Navrongo	2.00	1.75	2.54	1.75	1.91
Sunyani	–	1.62	1.68	–	–
Tarkwa	–	–	1.61	–	–
Wa	–	1.22	2.82	1.41	–
Yendi	–	1.07	1.78	1.46	–

–, no data

**Table 3:** Multiplicity of infection (mean MOI) for all the time points at the different ecological zones

Ecological zones	2003–2004	2005–2006	2007–2008	2010	2012–2013
Guinea savannah	2.00	1.32 ± 0.36	2.34 ± 0.54	1.53 ± 0.18	1.91
Forest	1.50	1.66 ± 0.05	2.10 ± 0.68	1.52 ± 0.17	1.59
Coastal savannah (1 site)	–	2.03	1.44	1.31	1.13
Ghana (pooled)	1.73 ± 0.35 (1.50–2.00)	1.55 ± 0.32 (1.07–2.03)	2.08 ± 0.62 (1.44–3.31)	1.49 ± 0.16 (1.31–1.75)	1.51 ± 0.39 (1.13–1.91)

–, no data

## Discussion

The parasite population structure of *P. falciparum* isolates from Ghana was determined using the diversity in the *msp2* gene over a decade. This genetic marker is recommended for genotyping parasites in antimalarial drug efficacy trials and parasite population structure analysis by the WHO [10–12]. The samples used in this investigation were collected from all the regions of Ghana which also represent the three distinct ecological zones in the country. The findings from this study revealed a high level of genetic diversity within the *msp2* gene, however there was lack of major differences in parasite variants across the country which may be as a result of a high level of gene flow due to human population movements (HPM) over the years. A recent genome-wide sequences analysis of isolates from two ecologically distinct areas in Ghana also showed the genetic structure of parasite populations as very similar [25]. The number of genotypes was 14 and 3D7 was the predominant allele from 2005 to 2010. Generally, the determined MOIs between ecological zones at the different time points and overall was not significant except for the 2007 isolates where a significant difference was observed among the nine sites.

*Msp2* block 3 is known to have a higher polymorphism and therefore provides very useful information in describing the diversity of parasites in a population compared to *msp1* and *glurp* [9, 13]. The study showed high polymorphism *msp2* gene and the predominant allelic genotypes detected had low to high frequencies which fluctuated at the different time points. This observation of predominant alleles is due to genetic recombinations that result in parasites with particular alleles having high frequencies and consequently a high level of biological fitness [26]. These findings are similar to observations made in Myanmar on the genetic diversity at the same locus and the observed fluctuations in allele frequencies of these predominant alleles over the years were attributed to selection by the hosts’ immune responses [27]. These immune responses are known to be strain-specific against recurrent parasites with same allelic antigens resulting in the differences in the prevalence of specific genotypes over time [27]. Of the two allelic families of the *msp2* gene, 3D7 was the predominant allele and this observation has also been made in several African and Southeast Asian countries [4, 9, 28–38]. Another report from Agyeman-Budu and colleagues who investigated parasite diversity in asymptomatic infections in the forest belt area of Ghana (Kintampo), showed a predominance of 3D7 over FC27 at a ratio of 4:1 in the dry season [39]. It is evident the 3D7 allelic family is the predominant *msp2* allele in high disease transmission areas.

There was generally no significant difference in the MOIs between the different ecological zones and also between time points when data were pooled; however, for the isolates from nine sites in 2007 showed variability in the MOIs. Although the population frequencies of the two *msp2* alleles (FC27 and 3D7) did not vary significantly among the nine populations in 2007 as a result of each local population having similarly wide spectrum of genotypes variation within each of these major types, the observed variation in MOIs estimates could not be due to differences in *msp2* allelic diversity locally (DJ Conway, personal communication). Whole genome sequence analyses show that parasite populations in different parts of West Africa have very similar genetic diversity [26, 40], and a comparison of different areas in Ghana has also shown that allele frequency distributions are very similar throughout the genome [25]. As these populations are well connected geographically, there is unlikely to be significant local divergence in allele frequencies of any parasite gene unless there have been significant differences in selection operating locally, as may be the case for drug resistance genes [17, 18]. It is known that the lack of differences between allele frequencies over time in a population is indicative of frequency equilibrium due to absence of selection which is controlled by frequency-dependent immune selection [41, 42]. This lack of significant variation in allele frequencies of *msp2* alleles as observed in our study over time has also been observed in parasite populations from the Gambia and Brazil [41, 43, 44].

The observation of lack of differences in parasite variants in all three ecological zones may be due to HPM across the country. For people living in the Guinea savannah ecological zone with seasonal transmission, during the dry season which could last for about 6 months, they become migrant workers who move to perennial transmission areas for economic reasons and return to farm their lands before the rains begin. As such they are carriers of parasites from their ecological zone to other zones and also carry parasites of the other zones back to their areas. HPM between rural and urban areas is the norm in the country for trading purposes and for visiting extended families which is an important cultural practice. Therefore, HPM greatly enhances the movement of parasite variants from one place to another which results in variants with minor differences in alleles of the *msp2* gene investigated across the country. The implication of the observed level of genetic diversity in parasite populations in high transmission areas as a result of genetic recombination poses a threat to the identification of antigenic epitopes for malaria vaccine design. Although extensive genetic diversity is a hindrance to vaccine development efforts, for people living in endemic areas, such diversity enhances the encounter with diverse parasite clones which in turn, help with the natural acquisition of immune responses against the multiple clones of the parasite and subsequent protection against disease symptoms.

Sequencing the polymorphic regions and microsatellite typing of the genetic locus investigated could provide deeper insight into the variations in gene sequences as detected by other studies [27, 29, 45], and therefore further analyses are ongoing which involves sequencing and microsatellite typing to reveal the genetic complexity of circulating parasites in Ghana.

## Conclusion

There was an immense genetic diversity in the parasite population in Ghana upon investigating the *msp2* gene. However, there was minimal variation or homogeneity in parasite populations across the country which may be due to gene flow from the effect of human population movements.

## Abbreviations

ACT, artemisinin-based combination therapy; ANOVA, analysis of variance; DNA, deoxyribonucleic acid; *glurp*, glutamine rich protein; HPM, human population movements; IPTp, intermittent preventive treatment in pregnancy; IRB, institutional review board; IRS, indoor residual spraying; LLINS, long lasting insecticide-treated nets; MOI, multiplicity of infection; *msp*1&2, merozoite surface proteins 1 and 2; NMCP, national malaria control programme; PCR, polymerase chain reaction; SMC, seasonal malaria chemoprevention; WHO, World Health Organization; WWARN, worldwide antimicrobial resistance network

## Additional files

Additional file 1: Table S1. Primer names and sequences for the detection of *msp2* alleles. (DOC 31 kb) Additional file 2: Table S2. Variation in genotypic mixedness of *Plasmodium falciparum* infections at the nine sites at different time points. (DOC 66 kb) Additional file 3: Table S3. Allele sizes and number of genotypes per infection for isolates collected in 2003–2004. (XLS 87 kb)
