# Supplementary material for: Genetic diversity of Plasmodium falciparum isolates from uncomplicated malaria cases in Ghana over a decade
Source: Parasit Vectors. 2016 Jul 26;9:416. doi: 10.1186/s13071-016-1692-1 (PMC4962487; doi:10.1186/s13071-016-1692-1)
Supplement: Additional file 1: Table S1. — Primer names and sequences for the detection of msp2 alleles. (DOC 31 kb) [file 13071_2016_1692_MOESM1_ESM.doc]

**Additional file 1: Table S1 Primer names and sequences for the detection of *msp2* alleles**

| **Primer name** | **Sequence** |
| --- | --- |
| **Primary**  **msp2-F** | 5'-ATGAAGGTAATTAAAACATTGTCTATTATA-3' |
| **msp2-R** | 5'-CTTTGTTACCATCGGTACATTCTT-3' |
| **Nested**  **msp2-FC27-F** | 5'-GCTTATAATATGAGTATAAGGAGAA-3' |
| **msp2-FC27-R** | 5'-TTTTATTTGGTGCATTGCCAGAACTTGAAC-3' |
| **msp2-3D7-F** | 5'-GCTTATAATATGAGTATAAGGAGAA-3' |
| **msp2-3D7-R** | 5'-CTGAAGAGGTACTGGTAG-3' |
