# Supplementary material for: Genetic diversity of Plasmodium falciparum isolates from uncomplicated malaria cases in Ghana over a decade
Source: Parasit Vectors. 2016 Jul 26;9:416. doi: 10.1186/s13071-016-1692-1 (PMC4962487; doi:10.1186/s13071-016-1692-1)
Supplement: Additional file 2: Table S2. — Variation in genotypic mixedness of Plasmodium falciparum infections at the nine sites at different time points. (DOC 66 kb) [file 13071_2016_1692_MOESM2_ESM.doc]

Additional file 2: **Table S2** Variation in genotypic mixedness of *Plasmodium falciparum* infections at the nine sites at different time points

|  |  |  | **Proportions (%) of *P. falciparum* clinical infections with each of the following numbers of different *msp2* alleles** | | | | | | |
| --- | --- | --- | --- | --- | --- | --- | --- | --- | --- |
| **Site** | **Year** | **n** | **1** | **2** | **3** | **4** | **5** | **6** | **7** |
| **Begoro** | 2005–06 | 23 | 43 | 52 | 0 | 4 | 0 | 0 | 0 |
|  | 2007–08 | 48 | 10 | 25 | 19 | 25 | 13 | 6 | 2 |
|  | 2010 | 11 | 64 | 27 | 9 | 0 | 0 | 0 | 0 |
|  |  |  |  |  |  |  |  |  |  |
| **Bekwai** | 2005–06 | 11 | 45 | 36 | 18 | 0 | 0 | 0 | 0 |
|  | 2007–08 | 44 | 30 | 36 | 27 | 7 | 0 | 0 | 0 |
|  | 2010 | 10 | 60 | 40 | 0 | 0 | 0 | 0 | 0 |
|  |  |  |  |  |  |  |  |  |  |
| **Cape Coast** | 2005–06 | 31 | 29 | 42 | 23 | 6 | 0 | 0 | 0 |
|  | 2007–08 | 27 | 69 | 23 | 0 | 8 | 0 | 0 | 0 |
|  | 2010 | 14 | 78 | 22 | 0 | 0 | 0 | 0 | 0 |
|  | 2012–13 | 38 | 82 | 18 | 0 | 0 | 0 | 0 | 0 |
|  |  |  |  |  |  |  |  |  |  |
| **Hohoe** | 2003–04 | 20 | 60 | 35 | 0 | 5 | 0 | 0 | 0 |
|  | 2007–08 | 37 | 27 | 38 | 24 | 11 | 0 | 0 | 0 |
|  | 2012–13 | 27 | 48 | 44 | 7 | 0 | 0 | 0 | 0 |
|  |  |  |  |  |  |  |  |  |  |
| **Navrongo** | 2003–04 | 17 | 24 | 59 | 12 | 6 | 0 | 0 | 0 |
|  | 2005–06 | 17 | 47 | 41 | 12 | 0 | 0 | 0 | 0 |
|  | 2007–08 | 41 | 15 | 32 | 34 | 20 | 0 | 0 | 0 |
|  | 2010 | 8 | 37 | 50 | 13 | 0 | 0 | 0 | 0 |
|  | 2012–13 | 68 | 31 | 54 | 7 | 7 | 0 | 0 | 0 |
|  |  |  |  |  |  |  |  |  |  |
| **Sunyani** | 2005–06 | 21 | 43 | 52 | 5 | 0 | 0 | 0 | 0 |
|  | 2007–08 | 40 | 50 | 35 | 15 | 0 | 0 | 0 | 0 |
|  |  |  |  |  |  |  |  |  |  |
| **Tarkwa** | 2007–08 | 18 | 44 | 50 | 6 | 0 | 0 | 0 | 0 |
|  |  |  |  |  |  |  |  |  |  |
| **Wa** | 2005–06 | 18 | 83 | 11 | 6 | 0 | 0 | 0 | 0 |
|  | 2007–08 | 38 | 29 | 11 | 18 | 34 | 8 | 0 | 0 |
|  | 2010 | 17 | 65 | 29 | 6 | 0 | 0 | 0 | 0 |
|  |  |  |  |  |  |  |  |  |  |
| **Yendi** | 2005–06 | 15 | 93 | 7 | 0 | 0 | 0 | 0 | 0 |
|  | 2007–08 | 39 | 44 | 36 | 16 | 5 | 0 | 0 | 0 |
|  | 2010 | 13 | 54 | 46 | 0 | 0 | 0 | 0 | 0 |
